# Complete Genome Sequence of Stenotrophomonas maltophilia Podophage Ptah

**DOI:** 10.1128/mra.00137-22

**Published:** 2022-03-14

**Authors:** Amelia Berg, Nathaniel Tate, James Clark, Tram Le, Ben Burrowes, Mei Liu

**Affiliations:** a Department of Biochemistry and Biophysics, Texas A&M University, College Station, Texas, USA; b Center for Phage Technology, Texas A&M University, College Station, Texas, USA; c BB Phage Consultancy, LLC, Georgetown, Texas, USA; DOE Joint Genome Institute

## Abstract

Stenotrophomonas maltophilia is an opportunistic pathogen demonstrating increasing drug resistance. Here, the genome of the T7-like S. maltophilia podophage Ptah is described. Its 42,593-bp genome is closely related to previously reported T7-like S. maltophilia podophages, including phage Ponderosa.

## ANNOUNCEMENT

Stenotrophomonas maltophilia is a Gram-negative bacterium that is found primarily in aqueous habitats and is able to cause respiratory infections in humans (1). With S. maltophilia demonstrating emerging drug resistance, the search for antibiotic alternatives such as phage therapy becomes increasingly relevant (2). Here, the isolation and characterization of S. maltophilia phage Ptah are described.

Phage Ptah was isolated, using S. maltophilia (ATCC 17807) as the propagation host, from a filtered (0.2-μm pore size) influent wastewater sample collected from the wastewater treatment plant in Beaumont, Texas, in September 2019. The host strain was routinely propagated aerobically at 30°C in tryptone nutrient (0.5% tryptone, 0.25% yeast extract, 0.1% glucose, 0.85% NaCl [wt/vol]) broth or agar. Isolation of the phage involved three rounds of plaque purification using the soft-agar overlay method (3). Phage genomic DNA was purified from polyethylene glycol (PEG)-precipitated phage particles from ∼8 mL phage lysate using by a Promega Wizard DNA cleanup system as described previously (4). Purified DNA was made into sequencing libraries using a Swift 2S Turbo kit with 300-bp inserts and was sequenced on an Illumina MiSeq system with paired-end 150-bp reads using 300-cycle v2 chemistry. The sequence reads were quality controlled using FastQC (http://www.bioinformatics.babraham.ac.uk/projects/fastqc) and trimmed using the FASTX-Toolkit v0.0.14 (http://hannonlab.cshl.edu/fastx_toolkit). Trimmed reads (107,842 reads in total) were assembled into a single contig with 76-fold coverage using SPAdes v3.5.0 (5). Sanger sequencing was performed on PCR products, amplifying the raw contig ends (forward primer, 5′-CCTGCAAGGCAGCTAGTGAT-3′; reverse primer, 5′-CCAGTCTCGCCATCATTGGT-3′), to verify the completeness of the genome, because the contig was randomly opened after the assembly. Annotation was done using the Center for Phage Technology (CPT) Galaxy-Apollo platform (https://cpt.tamu.edu/galaxy-pub) (6–8). Structural genes were predicted using GLIMMER v3.0 and MetaGeneAnnotator v1.0 (9, 10). tRNA gene predictions used ARAGORN v2.36 and tRNAscan-SE v2.0 (11, 12). InterProScan v5.48, BLAST v2.9.0, TMHMM v2.0, HHPred, LipoP v1.0, and SignalP v5.0 were used with default settings for gene function prediction (13–18). BLAST searches were performed against the NCBI nonredundant and UniProtKB Swiss-Prot (19) databases with a maximum expectation value of 0.001. Genome-wide DNA sequence similarity was calculated using progressiveMauve v2.4 (20). PhageTerm was used to predict phage termini from the raw sequencing reads (21). All tools were run with default settings unless otherwise specified.

**FIG 1:**
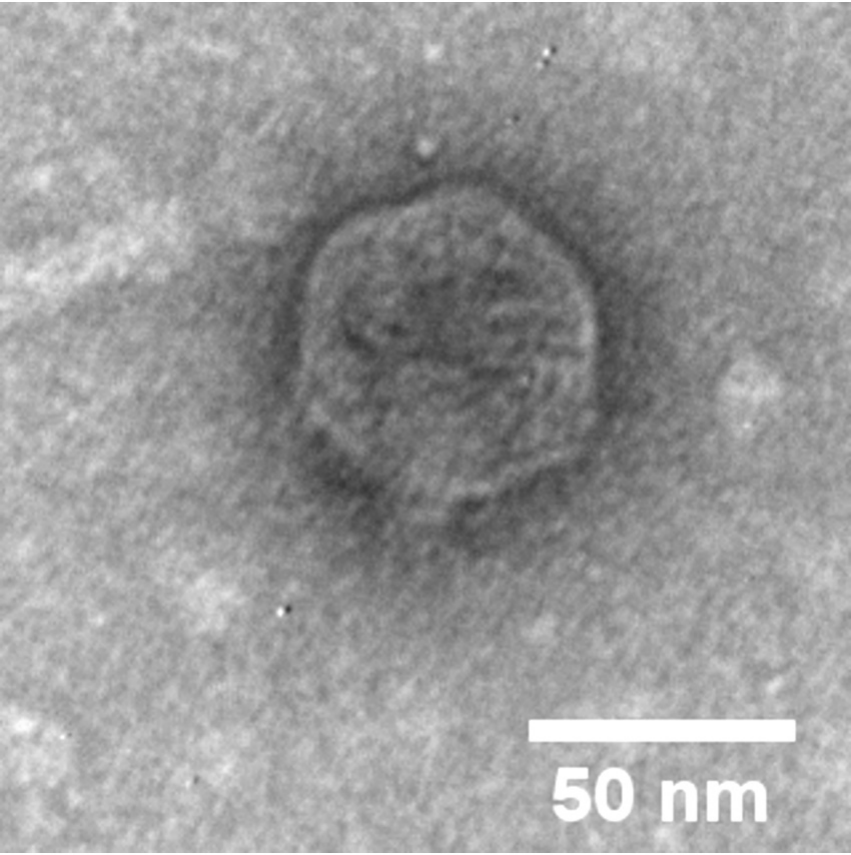
Transmission electron micrograph (TEM) of phage Ptah. Phage particles were diluted with TEM buffer (20 mM NaCl, 10 mM Tris-HCl [pH 7.5], 2 mM MgSO_4_) and captured on freshly glow-discharged, Formvar carbon-coated grids. The grids were stained with 2% (wt/vol) uranyl acetate and observed with a JEOL 1200 EX instrument at 100-kV accelerating voltage at the Microscopy and Imaging Center at Texas A&M University.

Ptah was identified as a podophage (Fig. 1) by negatively staining the sample with 2% (wt/vol) uranyl acetate and viewing it via transmission electron microscopy (TEM) at the Texas A&M Microscopy and Imaging Center. Phage Ptah has a 42,593-bp genome with a GC content of 61.8% and a coding density of 94.8%. In total, 56 protein-encoding genes were predicted, of which only 18 were assigned a predicted function. Analysis identified lysis genes, including a signal-arrest-release (SAR) endolysin, i-spanin, and overlapping o-spanin. No holin gene could be reliably identified, and only one tail fiber was identified with confidence. Genome comparison analysis revealed that Ptah is most closely related to S. maltophilia phage Ponderosa (GenBank accession number MK903280) (22), with 51 shared proteins (BLASTp; E value, <0.001) and 83.25% genome-wide nucleotide identity, as determined by progressiveMauve. Like phage Ponderosa, phage Ptah is T7-like in genome organization and size. The precise terminal repeat sequences could not be determined by PhageTerm.

### Data availability.

The genome of Ptah was deposited in GenBank with accession number MZ326854. The associated BioProject, SRA, and BioSample accession numbers are PRJNA222858, SRR14095248, and SAMN18495112, respectively.
